# Support in digital health skill development for vulnerable groups in a public library setting: perspectives of trainers

**DOI:** 10.3389/fdgth.2024.1519964

**Published:** 2025-01-13

**Authors:** Lucille M. B. Standaar, Adriana M. C. Israel, Rosalie van der Vaart, Brigitta Keij, Roland D. Friele, Mariëlle A. Beenackers, L. H. D. van Tuyl

**Affiliations:** ^1^ Department of Population Health and Health Services Research, Centre for Public Health, Healthcare and Society, National Institute for Public Health and the Environment, Bilthoven, Netherlands; ^2^ Department Organisation and Quality of Care, Netherlands Institute for Health Services Research, Utrecht, Netherlands; ^3^ Department of Tranzo, School of Social and Behavioral Sciences, Tilburg University, Tilburg, Netherlands; ^4^ Department of Public Health, Erasmus MC, University Medical Center Rotterdam, Rotterdam, Netherlands; ^5^ Research Group Technology for Healthcare, Centre of Expertise Health Innovation, The Hague University of Applied Sciences, The Hague, Netherlands

**Keywords:** digital health, health education, socio-economic factors, social services, older adults, health literacy, vulnerable populations, digital divide

## Abstract

**Introduction:**

The digitalization of healthcare poses a risk of exacerbating health inequalities. Dutch public libraries offer freely accessible e-health courses given by trainers. However, there is limited knowledge on whether these libraries successfully reach and support those in need. This study aimed to explore trainers’ perspectives on the challenges, successes, and potential improvements in digital health skill education in a library setting.

**Materials and methods:**

Trainers of the e-health course were interviewed. Topics included: the role of the library in digital health skills education, the successes and challenges in reaching groups with a low socioeconomic position, the perceived impact of the digital health skills education, and strategies for future improvement in digital health skills education. A deductive analysis based upon the interview guide topics was performed. A second inductive analysis was applied to identify underlying patterns. Coding was done independently and cross-checked. Codebooks and themes were determined in discussion with authors.

**Results:**

Three themes emerged. 1) Trainers’ services, skills and expertise: Trainers identified older adults, youth, people with low (digital) literacy, the unemployed, and people from non-native cultural backgrounds as the groups most in need of support. Trainers felt equipped to address these groups’ needs. 2) The libraries’ reach: improving engagement, perceived accessibility, and clients’ barriers: Despite trainers’ efforts to adjust the course to the target groups’ level of commitment, digital and literacy levels, and logistics, the digital health course predominantly engages older adults. Experienced barriers in reach: limited perceived accessibility of the public library and clients’ personal barriers. 3) Collaborations with healthcare, welfare and community organizations: Trainers emphasized that collaborations could enhance the diversity and number of participants. Current partnerships provided: reach to target groups, teaching locations, and referral of clients.

**Discussion:**

Trainers in public libraries recognize a various target groups that need support in digital health skill development. The study identified three challenges: accessibility of the digital health course, reach of the public library, and clients’ personal barriers. Public libraries have potential to support their target groups but need strategies to improve their engagement and reach. Collaborations with healthcare, welfare, and community organizations are essential to improve their reach to those most in need of support.

## Introduction

Rapid digitalization of healthcare imposes barriers in access to care for people with low digital health literacy or limited access to resources (1–3). Digital health, or e-health, is the use of information and communications technologies in healthcare to manage illnesses and health risks and to promote wellbeing (4). Digital health has a broad scope and includes the use of wearable devices, mobile health, telehealth, health information technology, and telemedicine (4)*.* Known barriers to use digital health are a lack of access to hard- and software, a lack of language literacy, digital health literacy, digital skills, and limited awareness of the existence and the benefits of e-health (5–9). Digital health literacy is defined by Norman et al. as “the ability to seek, find, understand, and appraise health information from electronic sources and apply the knowledge gained to addressing or solving a health problem” (10). This leads to a growing concern that the increasing adoption of e-health will lead to increased disparities in health (11, 12). The Digital Divide Model by Van Dijk describes the existence of a socio-economic gradient to the availability of resources that lead to access, use and derived benefit from new media, such as e-health (13). These resources are categorized in temporal, material, mental, social and cultural resources (13). According to the Digital Divide model and related research, policies and laws are countries’ instruments to redistribute public resources in an equitable manner to mitigate this divide (1, 13–20).

Scholars expect that the need for social services in a fast digitizing society and digitizing health care system will grow (21, 22). Public libraries have the opportunity to become key in facilitating education and support within a community setting increasing resilience to societal changes and improve inclusion of all citizens in modern society (23–25). International and national policies push the public library forward to facilitate support in the development of digital health literacy among citizens (26–34). Many studies underline the potential of libraries to educate citizens in digital skills and in health literacy, and increasingly in the combination of the two; digital health literacy skills (26, 28, 35–40). Libraries are viewed as accessible for anyone and well-rooted in society. Therefore, the library is considered a suitable avenue for reaching populations in need of support (18, 19).

Dutch public libraries are evolving from a source of written information towards an institute with a broad societal role, facilitating support in a variety of skills, including digital skills and accessing e-governmental services (31, 41–45). These developments show that the Dutch public library is a social services organization that is expanding their variety of services with the aim to help citizens to overcome challenges in current society. In this role, libraries predominantly focus on supporting people with limited language literacy, limited digital skills, in particular: older adults, parents with limited language skills, refugees and immigrants, and people with a lower socio-economic position (SEP) (46). Around 4 million Dutch citizens lack the language and numeric skills to participate in the knowledge-driven economy of the Netherlands (41, 42, 45). The public library estimates the level of language and numerical skills to be indicative for the level of digital skills (41, 42, 45). The Dutch library covenant (2021–2023) states the goal to reach 10%–20% of these citizens with support for the development of digital skills by employing effective interventions (42).

The Dutch government and the public library organization developed a program for digital inclusion (43, 44). Helpdesks [Informatiepunt Digitale Overheid] were opened in most libraries with the purpose to provide direct support in digital governmental, health and tax matters or refer clients to other forms of support offered by the library, such as courses or walk-in hours (43, 44). This study focuses on the digital health education provided in the public library, which is mainly facilitated via the trainer-led e-health course “DigiVitaler”. We previously showed that participants of the e-health course valued the content of the course and felt supported in their digital health skill development (47) and, that the participants were predominantly older adults (47). The general aim of the public library is to reach and educate a variety of people who need support in digital skill development most (46). To gain more insight into how the support in digital health skill development is realized in daily practice and if, how this support could be improved, trainers of the e-health course are interviewed.

This research aimed to gain insight into trainers’ perspectives regarding the challenges, successes, and possible future improvements in digital health skills education within the context of the Dutch public library. Results from this study provide grass-root level insights into what is needed to expand the role of the public library and the trainer as a valuable resource within the welfare and healthcare infrastructure in the context of digital health literacy.

## Materials and methods

### Setting

The organization DigiSterker develops courses for digital health skills, DigiVitaler and digital government skills, a.o. DigiSterker. Organizations that want to facilitate the courses have to purchase a license and trainers must follow a trainer education program (48). The public library organization has purchased the license of DigiVitaler for all public libraries to use (48). The course is available for public libraries to implement in their services since 2021 (49, 50). Each library needs to have a trainer that completed the trainer education program before offering the DigiVitaler course. The course DigiVitaler aims to enhance digital health literacy and is offered in 60 of 137 public library organizations in the Netherlands (2022) (49, 50). This course is structured into multiple classes, each covering a specific e-health topic, such as the patient portal, video consultations, and searching for online health information. Trainers introduce clients to the potential of e-health by providing hands-on experience with e-health applications in simulated digital environments. Libraries are free to decide what topics they offer and how the course is structured. There is no measurement of the level of digital skills before or after the course. In public libraries, participation in the course is free of charge.

### Data collection

A qualitative research design with semi-structured interviews was used. DigiVitaler trainers were interviewed regarding their daily practices in providing support for the development of digital health skills for people with a low SEP. The interviews took place in June and July 2022. The topics discussed included: (1) the role of the public library in digital health skills education, (2) the successes and challenges in reaching low SEP groups, (3) the perceived impact of the content of DigiVitaler, and (4) strategies for future improvement in digital health skills education. The interview guide was developed in collaboration with authors Lucille Standaar (LS), Adriana Israel (AI), Lilian van Tuyl (LvT), and Anita Suijkerbuijk (AS) and can be found in Supplementary File S1. In addition, the age, sex, level of education, job description and years of work experience of each participant were recorded. The level of education was determined according to the International Standard Classification of Education (ISCED): 0–2 corresponds with low, 3–4 with intermediate and 5–6 with a high level of education.

#### Recruitment

Trainers were eligible for participation in this study if they worked as a trainer for the course DigiVitaler within a public library. Contact information for the DigiVitaler trainers was provided to the researchers through DigiSterker, the developer of DigiVitaler. The trainers were approached via email, phone, or face-to-face as part of a previous study to inquire if they were interested in participating in this study. Prior to the interviews, the trainers received digital information about the study and were asked to sign an informed consent form. Participants were provided the opportunity to ask questions about the study and the interview procedure via phone or e-mail. Informed consent forms were collected via e-mail prior to the start of the interview. Participants received a 15 euro digital voucher for their participation. The interviews were conducted online via Microsoft Teams by LS and AI. All the interviews were recorded and lasted between 30 and 60 min. The content of the interviews was discussed among LS and AI after each cycle of six interviews. After fourteen interviews, no new information was retrieved and after discussion between LS and AI, it was concluded that data saturation was reached.

Fourteen trainers participated in the study. The participants were predominantly female (*n* = 10), with a mean age of 54.8 years (range of 31–62 years). This is in line with the gender and age distribution of all employees within public libraries, as 83% is female (2022) and 87% of employees fall in the age range of 30–60+ years of age (2022) (51). Eleven trainers were employed by the library, two were volunteers, and one was self-employed. The trainers were recruited from twelve different libraries across the Netherlands, covering both rural and urban municipalities. Most participants (*n* = 12) were highly educated. The participants were involved in education of digital skills for 8 years on average (range 1.5–25 years). All participants completed the trainer education program from DigiSterker, the developer of the course DigiVitaler. A detailed overview of the participants’ demographics is presented in Supplementary Table S2.

### Data analysis

The interview audio recordings were transcribed verbatim by a professional transcription service. Data were analyzed with the Codebook Thematic Analysis approach (52). Codebook thematic analysis allows for a structured process of coding, theme development and conceptualization in a multidisciplinary team while maintaining the approach of reflective thematic analysis to explore the perspectives of the trainers in-depth (53). First, transcripts were read for data familiarization. A codebook was created based upon the topics from the interview guide. The first two interviews were double coded by LS, AS, LT, and AI who then collaboratively defined and fine-tuned the codebook. After reaching consensus, the interviews were coded and cross-checked by LS and AI, using MaxQDA for data management. This deductive analysis was performed to create an overview of the data gathered. A second, inductive, analysis was performed to identify underlying patterns. The following topics were analyzed inductively: libraries’ services for digital health skills development, trainers’ guidance, trainers’ observations, target groups, collaborations, ideas for the future. The first 20% of the transcript segments per topic were inductively coded by LS and AI separately. After reaching consensus, the rest of the transcripts were coded and cross-checked by LS and AI. The inductive codes were arranged into candidate themes. Candidate themes were then further defined through discussion between all researchers to determine the final themes. An overview of the final themes and the underlying codes can be found in Supplementary Table S1. Quotes were translated by using DeepL and checked by a native English speaker.

### Ethical considerations

The study was declared to fall outside the scope of the Dutch Medical Research Involving Human Subjects Act by the Clinical Expertise Center of the Dutch National Institute for Public Health and the Environment (VPZ−559).Transcripts of the interviews and the informed consent forms are stored and protected at the Dutch National Institute of Public Health and the Environment.

## Results

**Figure 1:**
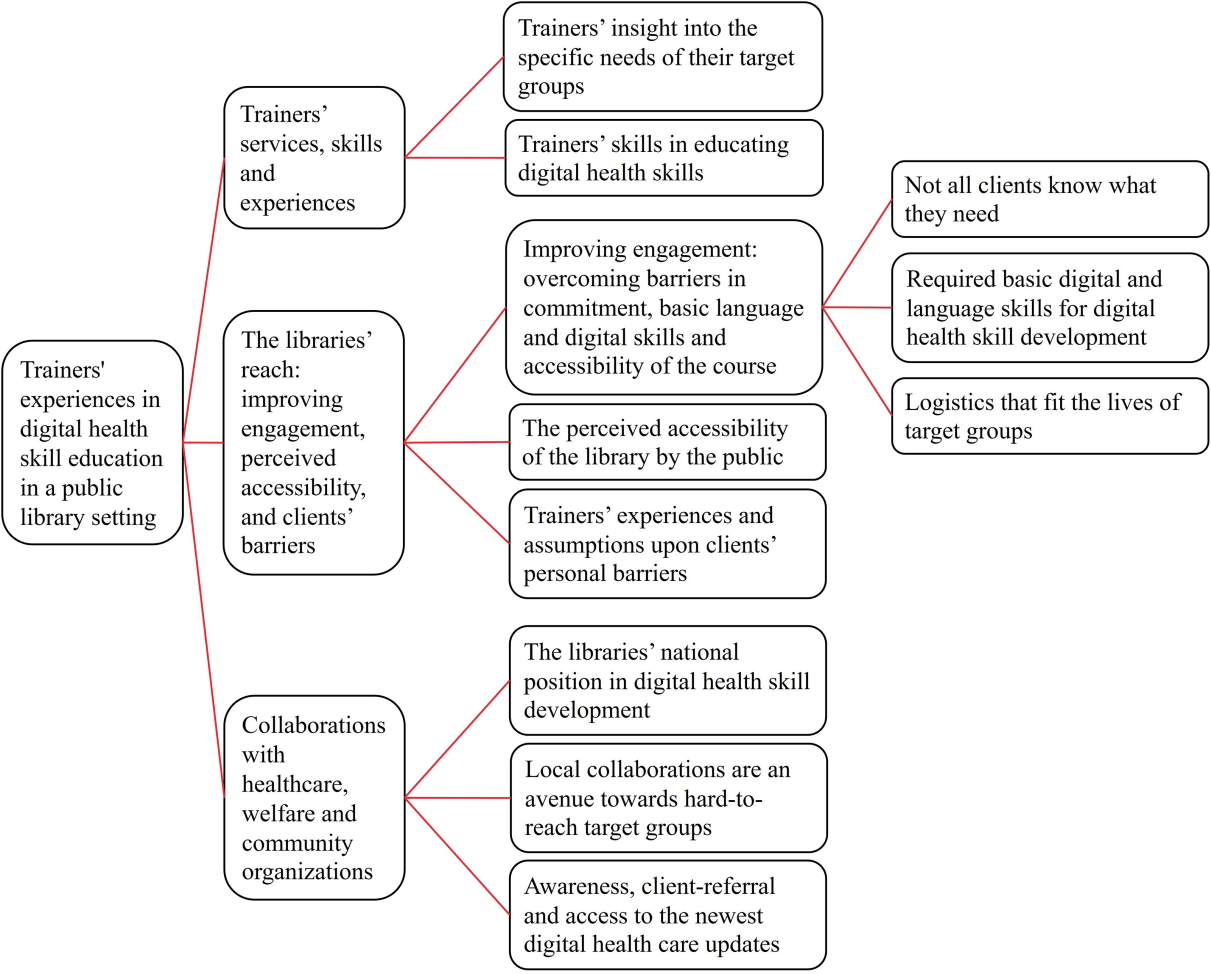
Overview of described themes and subthemes derived from the trainers’ experiences in digital health skills education in a public library setting. Data was collected via semi-structured interviews (*n* = 14). Interviews were held with Dutch trainers in digital health skills who taught in public libraries.

Three main themes were identified to describe the trainers’ experiences in providing support in digital health skills development in a public library setting: (1) Trainers’ services, skills and expertise, (2) The libraries’ reach: improving engagement, perceived accessibility and clients’ personal barriers, and (3) Collaborations with healthcare, welfare and community organizations. An overview of the themes and subthemes can be found in Figure 1.

### Trainers’ services, skills and experiences

#### Trainers’ insight into the specific needs of their target groups

The trainers felt that specific groups might need more assistance to acquire the skills necessary to access digital health. Most of the trainers have years of experience and worked with a diverse clientele in terms of age, skills, cultural backgrounds, and language proficiencies. Trainers expected the following populations to need support: people who are unemployed or who are not used to digital work, older people, persons with a migration background or people with low literacy skills, and the youth. The need for support was not perse related to socio-economic status according to the teachers. It was often mentioned that trainers expect different target groups to have different needs in accessing digital health.

‘A low-literate person may need help more often, but there are just people who have nothing to do with a smartphone or digital devices, but who are quite intelligent [..] they also need help. The immigrants who have just come to the country, who are still working on the language, but also need to know what DigiD is, [..] where they can go, what is on offer. So basically you are dealing with all layers.—Interview 13

‘Yes, newcomers. [..] Yes, with them I do have to make a comment, because they are digitally proficient. Because very often they come.. They have these nice mobile phones and so on, but they also have to be able to find their way within the Dutch infrastructure. And so we have to teach them that. - Interview 04

Youth and people with migration backgrounds were often seen as digitally skilled but were perceived to have less knowledge about how the (Dutch) governmental digital systems work. Additionally, some trainers experienced that the youth had less awareness of what safe use of the internet is and how important that is regarding their intensive participation in the digital world. Trainers observe that people with a migration background and people with limited literacy would benefit from developing their (Dutch) language skills to be able to fully participate in the digital health services.

“Youth are not so aware of what an app does and what information is stored. Who is the creator? Is it reliable? They are actually a little less concerned with that than would be good for them” - Interview 14

#### Trainers’ skills in educating digital health skills

Trainers reported to feel highly skilled and equipped to support the development of digital health skills among their clients. Most trainers explained that taking time and asking questions about their clients’ needs is important to be able to serve their clients best and adjust education efforts accordingly. Nevertheless, for each target group, different aspects of access to digital health are of importance and thus, a few trainers said to feel uncertain to best address the needs of some target groups. Their daily work includes providing courses, workshops, and directly accessible technical support to improve digital health skills of clients. Most trainers perceived the digital skills needed within digital health similar to other digital areas, as digital health often requires the use of a digital authenticator to log in, fill in forms or visit a website.

‘I now also start more from the student’s demand. In the beginning, of course, you have a fixed programme, and you want to run it. And then at a certain point I noticed that people had their own problems. So every course looks different and it kind of depends on what the course participants want.’—Interview 09

‘It’s mainly people who have been on welfare for a very long time. Yes, they are quite difficult to reach. And we know, we can't put our finger on that either, how digital-savvy are they actually?—Interview 04

Even though the trainers themselves feel well-equipped, they find that, in general, there is a lack of skilled and experienced workforce to strengthen their teams. The lack of skilled and experienced workforce poses a challenge on effectively educating their clients. Some trainers noted that they must depend on volunteers, which can occasionally result in education and support that does not fully meet the clients’ needs. Others mentioned that, to improve the quality of the libraries’ service, they would like to provide training in didactical skills and digital health for their volunteers and new library staff, but that they lack the stable financial funding for this type of development.

‘It’s also a choice for us to do that with professionals because you can also work with volunteers, you can also say, we have computers and people can just practice on their own but we do feel that we have to offer something more than that. We really have to guide people a bit in that, take them by the hand and also because of that, we spot what people are asking, what they are struggling with, and then of course we try to find an answer to that.’—Interview 01

‘There is still some subsidy for it now, but suppose that disappears, you have a chance that it will only have to be done by volunteers. That would be a shame, though, so I actually hope that the national management [..]will continue to pay attention to it—Interview 07

### The libraries’ reach: improving engagement, perceived accessibility, and clients’ barriers

Trainers experience that most clients who attended the course DigiVitaler were ethnic white female older adults of 60–80 years of age who were fluent in Dutch. Both successfully engaging diverse target groups in the digital health skill course and reaching target groups through the public library were experienced as challenging.

#### Improving engagement: overcoming barriers in commitment, basic language and digital skills and accessibility of the course

##### Not all clients know what they need

Trainers experience difficulty in balancing between the guidance needed for clients to learn new skills and the time-consuming effort that the development takes. Clients tend to overestimate their level of digital skills and rather commit to short courses. Most libraries had converted their multi-day courses into single workshops to address their clients’ needs. Even though, most trainers expressed that long term courses delivered to small classes are the most effective way to learn digital skills.

‘We thought of, shall we then extend our course? That we’ll make it, say, six classes? But that’s quite a long time span, that you're committing people and they don't want that so then you get that people aren't going to sign up again because the course is so long so it’s a bit of a balancing act as well, a bit of a commercial story, but how do you get people in?’—Interview 14

Regardless, most trainers felt that the digital health skills course improves clients’ confidence, skills and awareness in digital health, albeit it depends on the client how fast they progress in their skill development. Some trainers shared that people who only use the library for technical support tend to develop their digital skills less, as problems get solved for, and not by, clients.

‘Of course we have the digital government information point where people are really helped with what steps to take. In that you don't really teach people to become proficient themselves, that’s really more of a service and for me that’s a bit of the dividing line because at the IDO [Information Point Digital Government] you help people with a question they have. But if people want to learn how to do that themselves, they can go to the digital house’—Interview 03

##### Required basic digital and language skills for digital health skill development

According to the trainers, the e-health course demands a certain level of digital and language skills that not everyone possesses yet. Some participants mention that they assess the digital skill level of the client before admitting them to the digital health skills course. If the digital skill level is assessed as too low, the client is directed to a general course in digital skills. Some trainers mention that the course material would be difficult to teach to people with limited Dutch fluency and would direct these clients to language courses. Others mention that with the right tools and enough time, they would be able to teach digital health skills to non-native Dutch speakers.

‘You just ask some things. [..] Are they working with tablet? Are they working with a laptop? Or only on mobile? And what do they do then? [..] We have also recently developed a very short questionnaire that we ask people to fill in before they start taking courses. And then we can check whether, gosh, are they doing well or do they perhaps need to take another level [digital skills] lower or higher’—Interview 02

’Steffie’s [a website that provides videos explaining various topics in easy language] videos. [..] To reach people in a clear way also for example low-literate people and also even non-native speakers, because with some of the videos it is also possible that to see the text in your own language Arabic or Turkish. So that’s also how you reach that target group. So I also use those from time to time.’—Interview 09

##### Logistics that fit the lives of target groups

Trainers were aware of the importance of providing classes at times and places that suit their target groups. Some trainers offer classes according to the schedule and format (live/online) of their clients. One trainer mentioned that they offered the course at different times of the day to serve more target groups. Some trainers also offer their services in settings outside the library: community centers, in nearby villages without a library or during events from local associations such as older adults associations.

If you [as a younger adult] would then want to know something, you go and look it up in the evening [..]. And whether we have to do that in the evening physically, or whether we have to do that via Zoom or those kinds of methods, I'll leave that in the middle. But not everyone also has the need to then leave the living room in the evening and leave that hot cup of coffee because they are going to the library for a course.—Interview 08

‘We had a lot of courses during the day but we also deliberately made courses on evenings but that doesn't make much difference to the age group [..] the same people arrive there and we also switched evenings on occasion, saying, you know, is it because of Monday?’—Interview 06

‘We also went back into the neighbourhoods since last year, we are now in two neighbourhoods, socially disadvantaged neighbourhoods so to speak. And from September we will enter a third neighbourhood, where we will also have a drop-in point and walk-in consultations in community centres. To make it accessible to more people, because for many people the library is a complicated thing and not very accessible, whereas it should be, but it’s not in people’s minds to just walk into a library.’—Interview 03

#### The perceived accessibility of the library by the public

Most trainers expressed that they experienced difficulties in reaching the target groups that need support. All trainers felt that the public library is an organization that is and should be accessible for everyone. Some trainers found that the difficulties in reach originate in limited awareness of the libraries’ services or that the potential client was unable to commit to learning new skills due to limited time available or other priorities.

The perceived accessibility of the library by the public is mentioned as important for reaching the intended target groups. To improve the visibility of the course, trainers mention the use of various online and offline marketing methods, such as advertising in local newspapers, posters, flyers, word of mouth, and social media. Some libraries collaborate with older adults associations, general practitioners, pharmacies, and computer associations to use their network as an advertisement outlet. The trainers mentioned that most people that attend the course were made aware via newspaper advertisements or via local associations’ advertisement outlets.

‘I think you have to set it up as broadly as possible. And people who are actually confronted with it [digital healthcare] and don't know anything about it [digital healthcare], they will automatically come to you to start asking those questions’—Interview 08

Another perspective of the participants was that the library might be less accessible for certain target groups. Reluctance to visit the library may originate from perceived or experienced psychological, financial or logistic barriers. Trainers have mentioned that potential clients with limited literacy often avoid the library as they feel it is a place where they don't belong as they are unable to read. Others have mentioned that people may still have an old-fashioned image of the library; the library being a place for elites and where you can only read books. Some mention that for some people the library is too far away, travel is too expensive, or they experience discomfort leaving the familiarity of their neighborhood.

‘People with a migration background [..] they don't like to come here (public library in the city centre) either. [..]they say, it has to be here in the neighbourhood because it’s too far or I can't get there or they have to walk. It’s a combination of not being able to and finding it scary. Or that they have no money for it, but also because they find it scary. The neighbourhood is safe and especially for people with a migration background, who often stay in their neighbourhood and take their children to school’—Interview 03

#### Trainers’ experiences and assumptions upon clients’ personal barriers

Some trainers experience that especially the youth and middle-aged adults are difficult to engage in digital skills courses, even though they do ask for information at the helpdesk and are offered the option to attend a course. Some trainers assumed that shame for being less digitally skilled, lack of time or lack of motivation to invest in digital skills could explain this.

‘Yes, especially if we find that they [younger adults] could use some further training. And then we don't so much refer to that digital care, but more to the DigiD, working with DigiD and things like that. If we find that they are actually not that good with the computer at all, then it will also be the Klik & Tik [basic digital skill course]. [..]. Generally, they do take a leaflet with an offer, but I don't know whether they sign up then, you know. I think there is also a bit of shame there, I suspect. That they would rather go to one of these consulting hours just to ask for help again than to actually take a course.—Interview 07

Additionally, a few trainers also mentioned the importance of access to devices and awareness of the existence of e-health. The older adults and people with limited financial means often have less access to up-to-date devices. Even though libraries have devices available during the course, trainers feel that the course is less effective for people with little access to digital equipment. Clients may not have access to applications or websites due to outdated hardware or software. Some trainers mentioned that older adults are less aware of the possibilities of digital health because they might not be made aware as much as younger generations.

‘There are also people who don't have a laptop or a smartphone, who do have a computer at home, but it is either very slow or gathering dust because, of course, it’s no fun when it doesn't work properly or you can't use it much. They are often those discarded, you know, I have one left, mum, do you want it? That’s how it often goes in families, so there are also students who don't actually have all those devices and still want to know how it works but then, it just remains theory because they can't then put it into practice.’—Interview 01

‘A lot of people just don't know. Younger people know. Because they are made aware of it. So for example, my daughter knows about the app from the consultation office, the portal of the consultation office, because she has been made aware of it.’—Interview 12

One trainer mentioned that the digitalization of healthcare also involves a cultural change in how people interact with healthcare. Digital healthcare requires patients to take more initiative and, according to one interviewee, older adults and people from other cultures might not be used to this approach and need guidance to adjust to this new patient role.

‘It’s a big problem also culturally. So non-Western societies are much more hierarchical. My mother-in-law still has it very much, the doctor is still on a certain pedestal next to the mayor and the minister. And, for example, that is still the case in non-Western societies. So the idea that you can have your own direction in your health matters is completely insane to people.’—Interview 12

### Collaborations with healthcare, welfare and community organizations

Most trainers regarded the establishment of collaborations with various partners as a solution to improve their reach to target groups. Five benefits from collaborations were mentioned: enlarging advertisement reach, increasing client referral, facilitation of support in the living space of the target group, improving awareness about digital health skills education among healthcare professionals and citizens, and opportunities to exchange information between libraries and organizations. The mentioned partners were governmental organizations, health organizations, local hospitals or general practitioners, municipalities, community centres, (local) social services, older adults organizations, computer clubs, local leisure and sport clubs and schools.

#### The libraries’ national position in digital health skill development

The surge in (forced) digital health use during the COVID-19 pandemic revealed the gap in digital skills and digital health skills among the public, according to most trainers. As the need for digital health skills education was sudden, trainers felt that there was limited opportunity for the public library to fully establish their newly acquired position in the digital health field. Some trainers felt that due to the limited established position, healthcare organizations are unaware of the public libraries services or did not prioritize collaborations with the public library in the area of digital health skill development. Trainers also expressed that they felt that they didn't have the time or position to initiate larger scale collaborations. The enforcement of a network between the healthcare sector and the public library and a country-wide strategy for collaboration between the sectors is needed to improve the libraries’ position and reach, according to some trainers. Trainers shared that country-wide or larger collaborations could lead to improved awareness about the importance of digital health skills and the services of the library among patients and healthcare professionals. In return, trainers could benefit from being on top of the developments in the digital healthcare field. Additionally, these collaborations could provide opportunities for client referral to the library on a large scale.

‘Yes, much better cooperation with GPs and hospitals and maybe even health insurance companies. Because a lot of GPs don't yet know about the existence of DigiVitaler courses and they say ‘we don't have time.'[..] I would say, yes, how do you reach them all? Because we distributed flyers to the GP practices. We very actively tried to raise our profile. And they say, ‘no time, little time.’ Yes, so in that we would very much like to cooperate more. Also hospitals very much like to cooperate.. Yes, that would be very convenient. From the hospitals and general practices and specialists people referred, actively referred to us’—Interview 04

‘GPs and libraries, they have never had anything in common. So they have to start believing in each other first. You can't actually do that as an individual library. You have to do that nationwide.’—Interview 12

#### Local collaborations are an avenue towards hard-to-reach target groups

Trainers mentioned that by working together with local organizations, libraries can become part of a network within a neighborhood which makes their services more accessible to potential clients. Trainers provide different examples to how they reach youth, people living in low socio-economic neighborhoods, and older adults. One trainer elaborated upon a collaboration with local schools to reach youth by providing support at school. Multiple trainers mentioned collaborations with local organizations such as community centres and welfare organizations to facilitate education on locations within low SEP neighborhoods or in towns without a public library. Others mentioned collaborations with older adults associations and computer associations to use their advertisement avenues and presence at events to reach older adults. According to the trainers, collaborations with schools, older adults organizations, and computer associations were successful. These collaborations provided opportunities to reach target groups and to gain insight in the needs of the public libraries’ target groups. Some trainers mentioned that for libraries to be fully present in target groups’ neighborhoods, time and effort need to be invested in becoming locally known.

‘With us, that goes through information staff, who have lots of contacts with schools and with all kinds of youth organisations, and they recently had a brilliant pizza session. They invited young people to come and order pizzas, have a bite to eat with us and in exchange we will bother you with all kinds of questions, and that was actually quite a success, and that way you quickly get a feel for them.—Interview 14

‘In itself that is good, only there has to be some more awareness within those neighbourhoods too, that we are there. [..] Chatting, handing out flyers, then these things come to life as far as organisations are concerned [..]. And that just takes time and you have to keep repeating it, otherwise the information goes away again. So in itself I do think it works, but it could be much better.’—Interview 03

#### Awareness, client-referral and access to the newest digital healthcare updates

Some trainers reported that they are at the start of forming local collaborations with healthcare professionals, others mentioned that they wish to collaborate but have not yet found the time or the right contact person. The trainers found that collaborations with local healthcare organizations could improve the set-up of the digital health skills course by working together with healthcare professionals. According to the trainers, such collaborations would potentially provide opportunities for advertisement, client referral, and course locations.

‘We now have a pilot with two GP practices [..] that we get a notification if they [potential clients] have digital questions or problems with digital skills or with Dutch language, because we are connected to our language house.’—Interview 03

‘I am already trying to make contact with hospitals to see if maybe I can also give separate workshops about the hospital portal. And that the hospital then sends me patients or that I put information there in the form of a flyer. That they can go to the library if people just want to practice a bit more with the portal.’—Interview 09

## Discussion

In this study, the perspective of trainers on digital health skills education in public libraries was evaluated. The aim of this study was to gain insight into trainers’ perspectives regarding the challenges, successes, and possible future improvements in digital health skills education in the context of the Dutch public library.

Three main themes were identified that cover the experiences of the trainers of the digital health skills course offered by Dutch public libraries: (1) Trainers’ services, skills and experience, (2) the libraries’ reach: improving engagement, perceived accessibility, and clients’ barriers, and (3) collaborations with healthcare, welfare and community organizations. Trainers find the public library a fitting organization to facilitate support and education in digital health skills, however, to realize the public libraries’ potential, improvements are needed in the reach of the public library and the accessibility of the digital health skills course.

Trainers view the establishment of new collaborations as key in broadening the reach of the public libraries services and to realize the library's intention to reach diverse target groups that are in need of support (Theme 3) (42). By forming these collaborations, trainers expect that the public library will gain traction with a diverse clientele, improve awareness around digital health skills, and information exchange. Our previous research and other findings showed that motivation to learn digital health skills stems from awareness of e-health and its potential benefits, a sense of urgency because of a health issue or the feeling that participation in the digital world is inevitable (47, 54–56). Ramtohul describes that the existence of support in e-health use lowered the barrier for e-health use, but that a general interest in health, a health need, and saving costs were main triggers for end-users to use e-health (54). Results from this study show that trainers also assumed that feelings of shame about digital illiteracy, limited awareness and limited access to devices might cause barriers to seek support in digital health skill development. In our view, the healthcare sector, social welfare sector and the government should actively encourage and support citizens to adopt and adhere to e-health services, emphasizing the importance of developing digital health skills. Coetzer et al. identified a equivalent sentiment emerging from existing literature (57). Literature described the barriers of e-health use more often as individual-bound whereas suggested solutions to overcome these barriers were more often systemic-bound (57). Coetzer et al. argues that the barriers in e-health use are often framed as individual-bound, disregarding the role of systemic factors within these barriers (57). Consequently, Coetzer et al. concluded that access to e-health is a systemic responsibility, rather than only an individual one (57). Additionally, the literature showed that it is unclear how to translate these systemic solutions into real world settings (57). Similarly, the potential of the public library to improve (digital) health literacy is often mentioned in literature (18, 35, 36, 58–60); yet, evidence on how to realize the libraries’ potential is limited. Frank et al. describes how collaboration between the library and partners ensured access to devices, internet, and digital authentication via the library for vulnerable groups in rural America (61). Future research into collaborations between stakeholders from the healthcare sector, social welfare sector, and the government, as well as the capacities of stakeholders involved in digital health skill development, is needed. Such research could provide valuable insights into the development and enhancement of partnerships between the healthcare and social welfare sectors and what these partnerships could deliver to reduce digital health inequalities.

Trainers reflections upon who would benefit from an e-health course revealed that diverse target groups are at risk to possess limited digital health skills (Theme 1). Furthermore, trainers experienced that this risk lies in socio-economic, cultural and age related factors. Consequently, the trainers assume that different target groups require different approaches to improve digital health skills. Regardless of the trainers’ educational skills, awareness of their target groups needs and their intention to reach a wide population, predominantly older adults participate in the digital health skills courses (Theme 2). In reference to the Digital Divide model, trainers find that the course DigiVitaler resulted in the development of digital skills (mental resource), a formal point of contact that assists in digital health use (social resource) and the stimulus to form habits and stay up to date in digital health use (cultural resource) (13) for those who attend (Theme 2). Although the course is of quality according to the trainers, trainers experienced that some clients that did attend the digital health skills course have limited time and motivation to learn digital health skills. This causes trainers to provide workshops instead of multiple day courses to improve engagement, eventhough trainers assessed that this would be required for their clients to better develop digital health skills. The trainers mentioned that other and further course adjustments might be needed to successfully engage and educate different target groups.

Additionally, some trainers expressed concern that the perceived accessibility of the library might be a barrier for certain target groups, leading to the absence of target populations participating in the e-health course (Theme 2). The trainers mentioned that the prejudices regarding the library, the unfamiliarity with the library, and limited accessibility in terms of proximity and travel costs can pose a barrier to some target groups. Several researchers found similar findings (62–64). Results from Goedhart et al. describe that mothers with a low SEP view the library as an institute where people go who are already skilled in using the computer, and that they do not belong there (64). Evjen & Audunson and Goedhart et al. mention the importance of the integration of social services in one place to make the public spaces, such as the public library, more accessible (62, 64). A few scholars have provided practical insights into strategies that could improve libraries’ promotion of services, evaluation of their services and appropriation of their services to vulnerable groups (28, 59, 61, 65, 66). Dervin describes the need for collaborative approaches with the library consumer to improve the effectiveness of providing support in health information seeking and use (65). Two studies outside the library context provide examples of designing digital health skill education via co-creation with various stakeholders (67–69). Perestelo-Perez et al. described the co-creation process of massive open online courses (MOOC) to improve digital health literacy for specific groups such as youth, older adults, diabetes patients and pregnant or postpartum women (67). Whitney et al. provides examples of other education methods that were employed by libraries with the goal to reach and support a broader audience including children, older adults and people with a non-native language proficiency (66). These examples involved train-the-trainer principles, storytelling, and the use of new media such as virtual reality, gaming, and social media (66). Using such innovative methods can help reach and engage different target groups. Other studies describe different digital health skill development approaches. A literature study from Verweel et al. reports about the use of digital interventions to enhance digital health literacy skills from people with chronic illnesses, offering programs that often both target (digital) health literacy skills and the chronic condition at hand (70). These findings suggest that the integration of multiple goals in one program might be attractive for people who suffer from chronic illnesses. Dong et al. provided insight in the literature concerning digital health skills education for older adults, where both face-to-face and online educational approaches were described (71). Findings describe that theoretically underpinned, face-to-face education programmes with a duration of more than four weeks were more effective for improving e-health efficacy (71). Online methods were less effective but have other benefits according to Dong: flexibility in scheduling, opportunities to repeat lessons at home and improved access for those who already have sufficient digital skills and access to devices (71). Of the four identified online educational approaches one was co-created with the target group (69), one study recruited older adults with high digital skills (72), two were taught in a blended setting with help of a facilitator who provided technical assistance (73, 74). Findings suggests that both face-to-face and (blended) online educational approaches could be considered when designing digital health skill courses for older adults, the latter for those with sufficient digital skills and access to devices.

Our study had strengths and limitations. The interviewees were all experienced in digital skills education within the library setting and were recruited in different libraries across the country, ensuring a reliable information source to answer our research question. The interviews were conducted until data saturation was reached, indicating the robustness of our findings. A limitation of this study is that the findings are derived from a single perspective within the library organization and should be interpreted as such. The perspective of trainers provides a grass-root level insight to the successes and challenges the public library faces in digital health skills education. Future research into the insights from the public libraries’ leadership and relevant stakeholders such as the target groups, policy makers and health organization leaders would enrich the results from this study. Additionally, most of the interviewed trainers were employed by the library and had a high education level. This might have influenced the results of this study. Being highly educated and employed might influence the interviewees’ ability to understand the perceptions of those with less education or financial stability. However, there were limited opportunities to overcome this potential bias. At the time of data collection, the DigiVitaler course was new and predominantly implemented and taught by library staff with a job description that requires a certain level of education. Among our interviewees there were two volunteers and one self-employed trainer, yet this did not enhance the diversity in terms of educational background.

### Implications and directions for future research

Our findings showed that public libraries have potential to support Dutch citizens in the development of digital health skills. Our findings show that trainers have difficulties in establishing an effective outreach and education method. Regardless of their efforts to reach their intended target groups that ranged multiple age, socio-economic and cultural groups. Additionally, the trainers showed awareness of specific barriers for specific target groups, but potential solutions to increase engagement and reach lacked this detail. Literature reviews by Vassilakaki et al. and Barr-Walker identified similar challenges in American library settings (35, 36). These studies discussed the following solutions for creating effective health literacy programs: staff education, development of evaluation tools, beneficial partnerships and organizational leadership (35, 36). Our results and the literature reviews of Vassilakaki et al. and Barr-Walker (35, 36), imply that difficulties to effectively educate and reach diverse target groups is common among public libraries. These findings and the literature implicate two things. First, that the need for support in digital health skill development is not only caused by a socio-economic gradient, but that cultural and age related factors also play a role. Secondly, that there is limited insight into how public libraries can reach and educate their target groups and what the needs of target groups are in this matter. Most literature of the existing research within the library setting is focused on the American context. Findings outside this context indicate that co-creation of education materials with the target group, designing education materials that target multiple goals that are of importance for the target group and different online and offline approaches are potential avenues for improved reach and engagement (67–71). This presents opportunities to explore and research the needs and methods for reach and engagement for different target groups in other contexts. Our findings underlined that the perceived accessibility of the public library by the public was of importance according to the trainers. This factor was not identified in the literature reviews of Vassilakaki et al. and Barr-Walker (35, 36). Therefore, it would be interesting to create further understanding on how the image of the public library affects its perceived accessibility and the attendance of support in digital health skill development.

Our findings from a trainers’ perspective suggest that expanding library services to include support for digital health skills requires efforts at both local and national levels to fully realize its potential. Multiple models describe the process of interdisciplinary collaborations within the sphere of health and healthcare (75–77). Bronstein, Weinstein et al. and Corbin and Mittelmark all described a model with two levels of factors. First level factors describe the service users, practices, organizations and professions involved (75–77). Second level factors describe the process of interaction between different professions, people, organizations and practices (75–77). Several scholars have described the processes and in lesser extent the outcome of interdisciplinary collaborations between the healthcare sector and the social services sector. A literature review from Cameron et al. describes that organizational, cultural and contextual factors facilitate or hinder integrated collaboration efforts (78). Notably, studies reported the lack of appreciation of the contribution of different professions, the lack of understanding of the aims of integration and concern that the contribution of community health and social care services might be marginalized by the interests of the acute sector (78). Cameron finds that, although limited evidence is available, integrated collaborations can be effective upon health outcomes and reduce cost (78). Brewster et al. describes that within collaborations, high centrality of healthcare institution and co-sponsorship of healthcare institutions and social services for specific programs were correlated with high performing collaborations (79). The theoretical models and empirical results underline that successful integrated collaborations require careful design and demand at least (financial) commitment, clarity on aims and procedures and appreciation of all professions involved. At the organizational level, enhancing collaborative networks and improving coordination among stakeholders is essential to ensure that the support reaches those who it is meant for. Beyond trainers’ reflections on the potential roles of stakeholders in terms of reach, it is crucial to consider the extent in which public libraries can effectively enhance the digital health skills of target groups and fulfill their needs.

## Conclusion

Digitalization of healthcare requires large, diverse groups of people to invest in their digital skills. Digital health skills trainers within a public library setting estimate, based upon their previous experiences, that different target groups have different needs for digital healthcare use. Additionally, teachers identified their target groups’ needs to be socio-economic, cultural, and age-related. Challenges in providing support in digital health skill development are found in the accessibility of the digital health course, the reach of the public library, and clients’ personal barriers towards digital health and digital health skill development. From a trainers perspective, collaborations with healthcare, welfare, and community organizations are essential to improve the public's and stakeholders’ awareness of the libraries’ services in digital health skill development. This study shows that the public library has potential to become a partner in enabling a successful digitalization of the healthcare system. To realize this potential, research and practice should focus on identifying new methods to engage and educate different target groups. Furthermore, the enforcement of the network between the healthcare sector and the welfare sector is needed to realize access to digital health skill education for those who need it most.

## Data Availability

The raw data supporting the conclusions of this article will be made available by the authors, without undue reservation.
