# Supplementary material for: Support in digital health skill development for vulnerable groups in a public library setting: perspectives of trainers
Source: Front Digit Health. 2025 Jan 13;6:1519964. doi: 10.3389/fdgth.2024.1519964 (PMC11770011; doi:10.3389/fdgth.2024.1519964)
Supplement: Supplementary file 1 [file Supplementaryfile1.docx]

**Interview Guide: Trainers of the DigiVitaler Course**

**Introduction**
Thank you for allowing me to interview you today. My name is Lucille Standaar, and I am conducting research on the accessibility of digital healthcare. In this interview, we will focus on three main themes: the DigiVitaler course, the library's vision regarding the development of digital skills, and the library's role in fostering digital skills.

During the interview, you may stop at any point without needing to provide a reason. There are no right or wrong answers; I am interested in hearing your personal perspective. If there are questions you prefer not to answer, please let me know, and we will skip them.
Do you have any questions about the interview or the research?

**Informed Consent**: Confirmed?
**Recording**: Confirmed?

**Questions on Library Vision and Policy Regarding Digital Skills**

1. **What prompted the library to introduce the DigiVitaler course?**
   - Consider library policies, supply and demand, the impact of COVID-19 (e.g., DigiD [e-governmental identificator]), and the experimentation with new offerings.
2. **What is the library's vision for supporting the development of digital skills, including digital healthcare skills?**
   - Follow-up: How does this vision align with that of your local library, or alternatively, with the national library organisation’s vision?
3. **What role does the library envision for itself in supporting digital (health) skills?**
   - Is the library able to fulfill this role?
   - Why or why not? Are there areas for improvement?
   - Do you foresee changes in this role in the near future?
   - What resources are needed to better fulfill this role?
     - Collaboration with other organizations?
     - Increasing awareness of the library's new position?
     - Expanding available offerings?
4. **Who, in your opinion, needs support in developing digital skills, including digital healthcare skills?**
   - Follow-up: Does this relate specifically to DigiVitaler or other programs as well?
5. **Are these individuals being reached through the library's offerings in digital healthcare skills?**
   - Why or why not?
   - Perspective from the educator’s experience.
   - Does this concern DigiVitaler or other programs as well?
6. **What alternative methods could be used to reach more people in need of support?**

**Educator’s Perspective on Participants’ Experiences**

1. **From your observations as an educator, what motivates participants to join the course?**
2. **What feedback do participants provide during and after the course?**
3. **To what extent do participants actively engage in the course?**
   - Do they practice? How evident is their learning curve?
4. **In your opinion, what do participants gain from the course?**
   - What methods and materials are effective or ineffective?
     - Consider teaching methods, course materials, instructor support, and class size.
     - Do outcomes vary by participants’ skill level or demographic subgroups?
5. **What is the average level of digital skills among participants?**
6. **Do you feel that this course aligns with and supports the library's broader ambitions?**
   - If not, what improvements could be made?
7. **Are there other courses or forms of support available for using digital healthcare tools?**
   - What are the characteristics of these courses? Are there overlaps in target populations or differences in delivery methods (e.g., workshops, consultation hours)?
8. **Do you have suggestions for reaching people in a socially or economically vulnerable position to improve their digital skills?**

**General Questions About the Course**

1. **Since when has the library been offering the DigiVitaler course?**
2. **How frequently has the course been offered to date?**
   - How many times have you personally taught the course?
3. **What has the attendance been like?**
4. **Who typically participates in the course?**
   - I will present a series of statements, and I’d like you to indicate which category you find most applicable:
     - Participants are predominantly male, predominantly female, or evenly split.
     - What is the most common age group? Young adults / 30–50 / 50–70 / 70+  People with no migration background (native-born Dutch), individuals with a non-Western migration background, or individuals with a Western migration background?
     - Lower educated, higher educated, or evenly mixed?
     - Library members, non-members, or an even mix?
5. **How has the course been advertised?**
   - Examples: newsletters, local newspapers, posters, emails, other courses, or word-of-mouth
