# Supplementary material for: Support in digital health skill development for vulnerable groups in a public library setting: perspectives of trainers
Source: Front Digit Health. 2025 Jan 13;6:1519964. doi: 10.3389/fdgth.2024.1519964 (PMC11770011; doi:10.3389/fdgth.2024.1519964)
Supplement: Supplementary file 2 [file Table1.pdf]

Supplementary Table 1 – Overview of inductive themes and subthemes

Thema's	Clusters	No. of codes
Trainers' services, skills and experiences		
	Skills, knowlegde and experiences trainer	76
	Trainers' expertise and insight on development of digital health skills	60
	Target groups and needs	80
	Services	21
The libraries' reach: improving engagement, perceived accessibility, and clients' barriers		
	Accessibility of the organisation of the course	64
	Accessibility of the content of the course	43
	Public libraries' reach	58
	Dependent factor target group – location	6
	Dependent factor target group – values and believes	27
	Dependent factor target group – access to devices	4
Collaborations with healthcare, welfare and community organizations		
	Development, time and cost	39
	Collaborations	102
	Collaboration partners	34
	The position of the library within society – in relation to the public	5
	The position of the library within society – in relation to other organisations and the government	77
	Position of digitalisation within society – the public	59
	Position of digitalisation within society – the government and organisations	37
	Libraries' role	24
