# Supplementary material for: Support in digital health skill development for vulnerable groups in a public library setting: perspectives of trainers
Source: Front Digit Health. 2025 Jan 13;6:1519964. doi: 10.3389/fdgth.2024.1519964 (PMC11770011; doi:10.3389/fdgth.2024.1519964)
Supplement: Supplementary file 3 [file Table2.pdf]

Supplementary Table 2 – Overview demographics participants

No.	Age	Sexe (F/M)	Education[1]	Rurality public library[2]	Employment (years)	Type of employment	Years experience in digital skills education	Trained in digital skill education
1	51	F	High	Urban	6	Employed	6	Yes
2	41	F	Intermediate	Rural-urban	3	Employed	1	Yes
3	53	F	High	Urban	2	Employed	2	Yes
4	59	F	High	Rural-urban	20	Employed	6	Yes
5	75	M	High	Rural	10	Volunteer	10	Yes
6	56	F	High	Rural-urban	21	Employed	8	Yes
7	62	F	High	Rural-urban	42	Employed	25	Yes
8	67	M	High	Rural-urban	2,5	Volunteer	2,5	Yes
9	61	M	High	Urban	40	Employed	25	Yes
10	31	F	High	Rural-urban	5	Employed	2,5	Yes
11	61	F	Intermediate	Rural-urban	1,5	Employed	1,5	Yes
12	62	F	High	Urban	20	Employed	15	Yes
13	45	M	High	Rural-urban	3	Self-employed	3	Yes
14	43	F	High	Urban	6	Employed	6	Yes

[1] Educational background was categorized according to the International Standard Classification of Education (ISCED) (source)

[2] Rurality of the public library is derived from the rurality of the municipality the library is located in. Data retrieved from: Statistics Netherlands. "Gebieden in Nederland 2024" Retrieved 30-08-2024, from https://opendata.cbs.nl/#/CBS/nl/dataset/85755NED/table?searchKeywords=landelijk%20gebied
